# Russell-Like Bodies in Plant Seeds Share Common Features With Prolamin Bodies and Occur Upon Recombinant Protein Production

**DOI:** 10.3389/fpls.2019.00777

**Published:** 2019-06-26

**Authors:** Elsa Arcalis, Verena Ibl, Julia Hilscher, Thomas Rademacher, Linda Avesani, Francesca Morandini, Luisa Bortesi, Mario Pezzotti, Alessandro Vitale, Dietmar Pum, Thomas De Meyer, Ann Depicker, Eva Stoger

**Affiliations:** ^1^Department of Applied Genetics and Cell Biology, University of Natural Resources and Life Sciences, Vienna, Austria; ^2^Fraunhofer Institute for Molecular Biology and Applied Ecology IME, Aachen, Germany; ^3^Department of Biotechnology, University of Verona, Verona, Italy; ^4^Institute of Agricultural Biology and Biotechnology, CNR, Milan, Italy; ^5^Department of Nanobiotechnology, University of Natural Resources and Life Sciences, Vienna, Austria; ^6^Department of Plant Biotechnology and Bioinformatics, Ghent University, Ghent, Belgium; ^7^VIB Center for Plant Systems Biology, Ghent, Belgium

**Keywords:** molecular farming, recombinant protein, protein bodies, electron tomography, subcellular targeting

## Abstract

Although many recombinant proteins have been produced in seeds at high yields without adverse effects on the plant, endoplasmic reticulum (ER) stress and aberrant localization of endogenous or recombinant proteins have also been reported. The production of murine interleukin-10 (mIL-10) in *Arabidopsis thaliana* seeds resulted in the *de novo* formation of ER-derived structures containing a large fraction of the recombinant protein in an insoluble form. These bodies containing mIL-10 were morphologically similar to Russell bodies found in mammalian cells. We confirmed that the compartment containing mIL-10 was enclosed by ER membranes, and 3D electron microscopy revealed that these structures have a spheroidal shape. Another feature shared with Russell bodies is the continued viability of the cells that generate these organelles. To investigate similarities in the formation of Russell-like bodies and the plant-specific protein bodies formed by prolamins in cereal seeds, we crossed plants containing ectopic ER-derived prolamin protein bodies with a line accumulating mIL-10 in Russell-like bodies. This resulted in seeds containing only one population of protein bodies in which mIL-10 inclusions formed a central core surrounded by the prolamin-containing matrix, suggesting that both types of protein aggregates are together removed from the secretory pathway by a common mechanism. We propose that, like mammalian cells, plant cells are able to form Russell-like bodies as a self-protection mechanism, when they are overloaded with a partially transport-incompetent protein, and we discuss the resulting challenges for recombinant protein production.

## Introduction

Seeds are used as production hosts for recombinant pharmaceutical proteins because they are well-adapted for the synthesis and storage of complex proteins. Indeed, seed storage proteins are synthesized by the secretory pathway and their folding, and modification is supported by a complex orchestra of folding helpers in the endoplasmic reticulum (ER). The endomembrane system of seeds is devoted to protein accumulation, and to this purpose, it comprises specialized compartments of the endomembrane system, such as protein storage vacuoles (PSVs) and ER-derived protein bodies (Herman and Larkins, 1999; Shewry and Halford, 2002). Seeds are remarkably flexible in terms of their protein content and reducing the abundance of one protein is often compensated by increasing the abundance of others through a rebalancing mechanism, indicating plasticity within the endomembrane system (Vogel et al., 1978; Krishnan et al., 2007; Kawakatsu et al., 2010; Kawakatsu and Takaiwa, 2010; Schmidt et al., 2011; Takaiwa et al., 2018).

There are many examples of high yields of recombinant proteins in seeds (Boothe et al., 2010; Takaiwa et al., 2017). Among the cereals, human serum albumin has been expressed at levels exceeding 10% of total soluble protein (TSP) in rice (*Oryza sativa*) grains (He et al., 2011), and industrial enzymes have been produced at up to 30% of TSP in maize (*Zea mays*) grains (Devaiah et al., 2013). In dicotyledonous species, human apolipoprotein accumulated to high levels in safflower (*Carthamus tinctorius*) seeds (Nykiforuk et al., 2011), human growth hormone has been expressed at high levels in soybean (*Glycine max*) seeds (Cunha et al., 2011), and a murine single chain antibody has been produced in *Arabidopsis thaliana* at levels up to 36% of TSP (De Jaeger et al., 2002).

Although such high levels of recombinant protein synthesis presumably place great demands on the host, there have been only few reports of adverse effects on seed physiology, morphology, or yield, confirming a generally high degree of flexibility and adaptability of the protein synthesis machinery and the endomembrane system. In cereals such as maize and rice that accumulate large amounts of endogenous storage protein in ER-derived protein bodies, in some cases, minor distortions of protein body morphology have been detected by electron microscopy upon the expression of recombinant proteins (Yang et al., 2012; Peters et al., 2013; Wang et al., 2013). Other studies have found that recombinant protein synthesis may correlate with an ER stress response (Wang et al., 2015) or cause specific grain phenotypes, such as an opaque endosperm (Oono et al., 2010; De Wilde et al., 2013). The immunolocalization of a recombinant antibody fragment in *A. thaliana* seeds revealed the aberrant localization of a proportion of KDEL-tagged antibody in the periplasmic space and in ER-derived compartments delimited by ribosome-associated membranes (Van Droogenbroeck et al., 2007). Similar observations have been reported with other antibody fragments, both with or without the KDEL tag (Loos et al., 2011; De Meyer and Depicker, 2014). In some cases, the formation of ER derived organelles was accompanied by the mislocalization of native proteins, indicating that the normal protein-sorting mechanisms were disturbed (Van Droogenbroeck et al., 2007; Loos et al., 2011; De Meyer and Depicker, 2014).

Similar ER-derived structures containing secretion-incompetent proteins have been described in mammalian cells as a structural hallmark of ER storage diseases (Lomas, 2005; Gooptu and Lomas, 2009; Perlmutter, 2009; Ronzoni et al., 2010) and as a strategy to segregate insoluble proteins that might otherwise disrupt the secretory pathway (Valetti et al., 1991; Kopito and Sitia, 2000; Miranda et al., 2004; Granell et al., 2008; Ito et al., 2012). These are called Russell bodies (RBs) or ER-derived protective organelles (ERPOs) and have been attributed the role of an inducible “SOS” compartment that results from the condensation of abnormal and/or abundant proteins for which the rate of synthesis exceeds the combined rates of folding and degradation in the early secretory compartment (Valetti et al., 1991; Granell et al., 2008; Ito et al., 2012). It is unclear whether the formation of RBs and similar structures is pathogenic or protective, but there is evidence that they reduce the toxic effects of improperly folded proteins and prolong cell survival, thus protecting the cell by transferring insoluble material from the ER into physically separate organelles (Granell et al., 2008; Ito et al., 2012). The classic RBs in multiple myeloma cells were described as intracellular structures containing condensed immunoglobulins that are insoluble in non-ionic detergent and that can neither be secreted nor retrotranslocated and degraded by the ubiquitin-proteasome system (Russell, 1890; Hsu et al., 1982; Valetti et al., 1991). However, Russell-like bodies can also be induced by other types of proteins and in non-lymphoid cell types, promoted by mutations that interfere with protein folding or by environmental stress (Carlson et al., 1989; Lomas et al., 1992; Kim and Arvan, 1998; Mattioli et al., 2006; Yam et al., 2007). RBs were also observed in CHO cells producing recombinant antibodies (Hasegawa et al., 2014, 2017; Reinhart et al., 2014;). Similarly, in yeast cells, the expression of recombinant secretory proteins frequently causes an imbalance between the protein load and the ER folding capacity, resulting in the induction of ER stress and aggregate formation (Umebayashi et al., 1997; Yu et al., 2017).

In plants, the deposition of condensed material in distinct ER-derived structures has primarily been observed as part of physiological programs required for seed maturation (Herman and Larkins, 1999; Galili, 2004). Parallels between the formation of RBs and the sequestration of cereal storage proteins in ER-derived prolamin bodies (PBs) have been pointed out (Kopito and Sitia, 2000; Vitale and Ceriotti, 2004; Ronzoni et al., 2010), suggesting that both processes are based on a similar principle operating in plant as well as mammalian cells.

Previously, we described the production and localization of three recombinant pharmaceutical proteins in *A. thaliana* seeds: the 65-kDa isoform of glutamic acid decarboxylase (GAD67/65), pro-insulin, and murine interleukin-10 (mIL-10), each modified by the addition of a signal peptide for translocation into the ER, and a C-terminal KDEL sequence for ER-retention (Morandini et al., 2011). Although this signal combination usually leads to the expected ER-localization of a recombinant protein in vegetative tissues, in seed storage tissues accumulation in the PSVs is often a dominant localization of recombinant proteins, even when these are expected to be secreted or to be retained in the ER *via* the H/KDEL mechanism (Arcalis et al., 2013; De Meyer and Depicker, 2014). GAD67/65 and pro-insulin seem subjected to this dominant process of PSV delivery: Pro-insulin accumulated in PSVs, GAD67/65 was detected in PSVs and in the ER lumen (Morandini et al., 2011). However, despite sharing the same targeting sequences, mIL-10 mainly accumulated in newly formed membrane-bound compartments (Morandini et al., 2011). Here, we confirm their ER-origin and we demonstrate by 3D electron microscopy that the structures have a spheroidal shape. We show that mIL-10 contained in the ER-derived structures is insoluble in non-ionic detergent, thus resembling the situation with classic RBs. Plant cells containing these structures are viable, and seed germination is only slightly delayed. We conclude that, like mammalian cells, plant cells generate Russell-like bodies upon ER-stress triggered by large amounts of particular proteins accumulating in the secretory pathway.

## Materials and Methods

### Plant Material and Growth Conditions

*Arabidopsis thaliana*, ecotype Columbia 0 (Col-0) was used. Plant germination and growth conditions on sterile nutrient agar medium were as described by Hauser et al. (1995).

The generation and quantitative characterization of transgenic *A. thaliana* lines producing mIL-10 and GAD67/65 have been described before (Morandini et al., 2011). We selected three lines accumulating soluble mIL-10 at different levels (0.3, 0.14, and 0.05 mg/g seed dry weight) and used a line accumulating GAD67/65 (3 mg/g seed dry weight) as a control. *A. thaliana* lines producing scFv-Fc antibodies MBP-10, HA78, and EHF34 at levels between 7 and 13% of TSP have been described before (Van Droogenbroeck et al., 2007). Homozygous seed stocks were used for all lines. The *A. thaliana* marker line expressing the integral membrane GFP fusion with a C-terminal KKXX signal was kindly provided by D. Jones (Benghezal et al., 2000).

For the induction of ectopic DsRed-zein bodies, we used the previously described construct SP-DsZein (Hofbauer et al., 2014), in which tetrameric DsRed was joined *via* a (GGGS)2 linker to residues 4–93 of the mature 27 kDa γ-zein protein. A plant codon-optimized signal peptide sequence derived from a murine antibody was added at the N terminus to direct the protein into the secretory pathway. The synthetic fusion sequence was transferred to the modified binary expression vector pTRA vector under the control of the CaMV 35S promoter with duplicated enhancer. *A. thaliana* cv. Columbia plants were transformed using the floral dip method with *Agrobacterium tumefaciens* strain GV3101 (pMP90RK) carrying pTRA (Clough and Bent, 1998). The resulting plants were tested for transgene integration by PCR, and PCR-positive plants were self-crossed. Homozygous T4 seeds were used for analysis and for crossing with the best-performing mIL-10 line.

Supplementary Figure S1 provides overview schemes of all transgene cassettes used in the present study.

### Electron and Fluorescence Microscopy

Mature non-transformed control seeds and seeds expressing recombinant mIL-10 or GAD67/65 were fixed and processed as previously described (Morandini et al., 2011). Ultrathin sections mounted on grids for electron microscopy or thin sections mounted on glass slides for fluorescence microscopy were pre-incubated in 5% (w/v) BSA in 0.1 M phosphate buffer (pH 7.4) and incubated with a rabbit anti-mIL-10 antiserum (Acris Antibodies GmbH), a rabbit anti-GAD65 (Enzo), or a rabbit anti-DsRed (Takara-Clontech), diluted 1:100 in 0.1 M phosphate buffer (pH 7.4) for 2 h at RT. The sections were then incubated with a donkey anti-rabbit antiserum labeled with 10-nm gold particles diluted 1:50 in 0.1 M phosphate buffer (pH 7.4) for 1 h at RT for electron microscopy (FEI Tecnai G2 transmission electron microscope) or with Alexa Fluor^®^ 546 [1:50 in 0.1 M phosphate buffer (pH 7.4) for 1 h at RT] for fluorescence microscopy (Leica DM5500B). At least three samples per line, each containing a minimum of five seeds, were analyzed.

### Confocal Microscopy

The GFP-KKXX marker was crossed into the line expressing the highest levels of mIL-10, and the mature seeds were incubated overnight (16 h) at 4°C on a wet filter paper, before removing the seed coat and viewing by confocal microscopy (Leica TCS SP5). GFP was excited at 488 nm, and fluorescence emission was monitored at 503–530 nm, whereas DsRed was excited at 561 nm, and fluorescence emission was monitored at 573–642 nm. PSV autofluorescence was excited at 405 nm. Images produced by the Leica LAS software were processed using ImageJ and Adobe Photoshop CS5. At least two samples per line, each containing a minimum of five seeds, were analyzed.

### Electron Tomography

*A. thaliana* embryos were excised from mature seeds (imbibed in water for 16 h at 4°C), submerged in 7 mM Tris buffer (pH 6.6) containing 140 mM sucrose and 7 mM trehalose and mounted on 200-μm planchettes. The samples were frozen under high pressure, freeze substituted in 5% water, 0.2% OsO_4_ and 0.1% uranyl acetate, and infiltrated into agar 100 resin for 3 days. Semi-thick sections (300 nm) from embedded transgenic embryos were imaged using a FEI Tecnai G2 transmission electron microscope at 200 kV. Single-axis tilt-series were recorded from −68 to +68° using tilt increments of 5° in tilt angles up to ±50° and 1° from ±50 to ±68°. Tomograms were calculated by back projection using the etomo interface of the IMOD software package (Boulder Laboratory of 3D Electron Microscopy of the Cell, University of Colorado at Boulder). Tomogram analysis and 3D model reconstruction were based on the 3dmod graphic module of the IMOD software package.

### Sequential Protein Extraction and Immunoblot Analysis

Twenty microgram of mature wild-type and homozygous transgenic seeds, as well as corresponding seeds 3 days after germination (DAG), was ground in liquid nitrogen and extracted twice with hexane (1:50 w:v), and then in 30 volumes (600 μl) of freshly prepared buffer (50 mM Tris-HCl (pH 7.8), 200 mM NaCl, 5 mM EDTA, 0.1% (v/v) Tween-20). After centrifugation for 5 min at 4°C, 14,000 g the supernatant was collected, the pellet was re-extracted three times for 5 min with the same buffer, and the supernatant from the third wash was kept as a control. Finally, the residual proteins were extracted from the pellet with the same volume of buffer complemented with 8 M urea and 5% 2-mercaptoethanol as appropriate. Equal amounts of extract (10 μl) were separated by sodium dodecylsulfate polyacrylamide gel electrophoresis (SDS-PAGE) on 12% (w/v) gels prior to blotting. The membranes were blocked with 4% (w/v) non-fat milk and incubated at 4°C with the appropriate primary antibodies (Morandini et al., 2011). For relative protein quantification blots were incubated with rabbit anti-mIL-10 Ab (1 mg/ml; PP007P1, Acris Antibodies GmbH) at 4°C overnight (1:5,000 in PBS-Tween-20 0.05%), followed by anti-rabbit IgG HRP-linked Ab (#7074, Cell Signaling Technology) at room temperature for 1 h (1:10,000 in PBS-T). For chemiluminescence detection, membranes were incubated with ClarityTM Western ECL Blotting Substrate (BIO-RAD), and chemiluminescence was documented by the Fusion Solo S (Vilber Lourmat) imager. AP-conjugated anti-human IgG antibodies (Promega S382B) were used to detect the scFv-Fc antibodies.

Relative quantification was carried out using the quantification module implemented in the Fusion Solo S software and is based on five blots using five independent protein extracts of homozygous seeds.

### RNA Extraction and Quantitative Reverse Transcription Polymerase Chain Reaction Analysis

Arabidopsis Columbia wild-type, mIL-10 and GAD67/65 expressing plants were grown at 25°C under a 16/8 h light/dark cycle. All plant lines used in the study were grown at the same time under the same experimental conditions. Homozygous mature seeds of the same line were pooled per tray (around 70 individuals), and quantitative reverse transcription polymerase chain reaction (RT-PCR) analysis was based on cDNA isolated from three separate seed samples per line. RNA was extracted from dry mature seeds (50 mg) using SpectrumTM Plant Total RNA Kit (Merck). Two microgram of total RNA was treated with DNaseI (ThermoFisher Scientific) before being subjected to reverse transcription (M-MuLV H plus Reverse Transcriptase, Promega). A DNaseI treated, non-reverse transcribed sample was included to test for DNaseI treatment. cDNA templates were run in technical triplicates. qPCR using SYBR green detection (5× HOT FIREPol^®^ EvaGreen qPCR Mix Plus wo ROX, Solis BioDyne) was carried out in a Rotor-Gene 3000 (Corbett Research) with the running profile: 95°C for 12 min followed by 40 cycles of 95°C for 5 s, 55/59°C (*BIP3*/*LSM4*) for 5 s, 72°C for 20 s. The run was completed by a melting analysis from 65 to 95°C rising by 1°C steps. Forward and reverse primers for *BIP3* were taken from Wang et al. (2015). Small nuclear ribonucleoprotein family protein *LSM4* (AT5G27720) was used as internal control gene, using primers 5′ACCACCAGGTGTTGGACGTG3′ and 5′CATCAACCACGGCCGCGAC3′. Data were analyzed using the Rotor-Gene 6 software (Corbett Research). PCR efficiencies for BIP3 (*E* = 1.78) and LSM4 (*E* = 1.78) are derived from the slope of a simultaneously run serial dilution of respective PCR templates. *BIP3* levels are normalized to *LSM4* as internal control gene, and expression values are given as 1/(E-ct BIP3/E-ct LSM4). Error bars depict ±standard deviation of the sample mean, which was calculated based on three replicates (measurement of three cDNAs derived from separate homozygous seed samples for each line). To determine statistical significance a Student’s *t* test was performed (*p* < 0.01 for all comparisons; *n* = 3, two-sided test). Plants compared in this experiment were grown at the same time under the same experimental conditions.

## Results

### Different Fractions of mIL-10 Can Be Distinguished in *A. thaliana* Cotyledons

**Figure 1:**
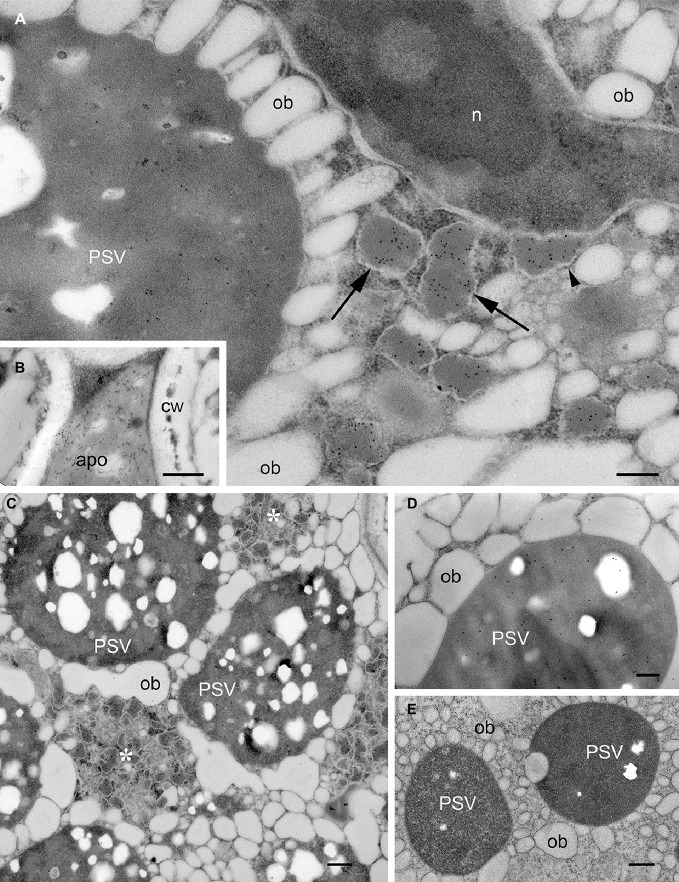
Localization of mIL-10 and GAD 67/65 in *A. thaliana* seed cotyledon cells. Representative electron micrographs showing the best-performing lines for expression of mIL-10 **(A–C)**, GAD 67/65 **(D)**, and **(E)** wild-type seed. Gold particles are visible in protein storage vacuoles (PSV), the apoplast (apo), and especially in membrane-delimited structures (arrows), surrounded by ribosomes (arrowhead) and forming clusters within the cytoplasm (*). No comparable ER-derived vesicular structures were observed in wild-type seeds. Cell wall (cw), nucleus (n), and oil bodies (ob) are also indicated. Bars = 0.25 μm. Electron micrographs showing the lines producing mIL-10 at lower levels are provided in Supplementary Figure S3.

The subcellular localization of recombinant mIL-10 was investigated by electron microscopy using three transgenic lines accumulating mIL-10 at different levels (0.3, 0.140, and 0.05 mg/g, respectively; Morandini et al., 2011). In the line that accumulates highest amounts, mIL-10 was in part secreted but mainly found in large ER-derived vesicles (Figures 1A,B, arrows), as previously reported (Morandini et al., 2011). Scattered labeling could also be identified in PSVs of transgenic cotyledonary cells (Figures 1A,C). The ER-derived vesicles containing mIL-10 were 200–500 nm in size and tended to form clusters within the cytoplasm. The ultrastructure of cells containing such bodies was different from that of wild-type cells: oil bodies were often partially fused and showed a wider size range than those in wild-type cells (Figures 1C,E). GAD67/65 was mainly detected in PSVs (Figure 1D; Morandini et al., 2011).

**Figure 2:**
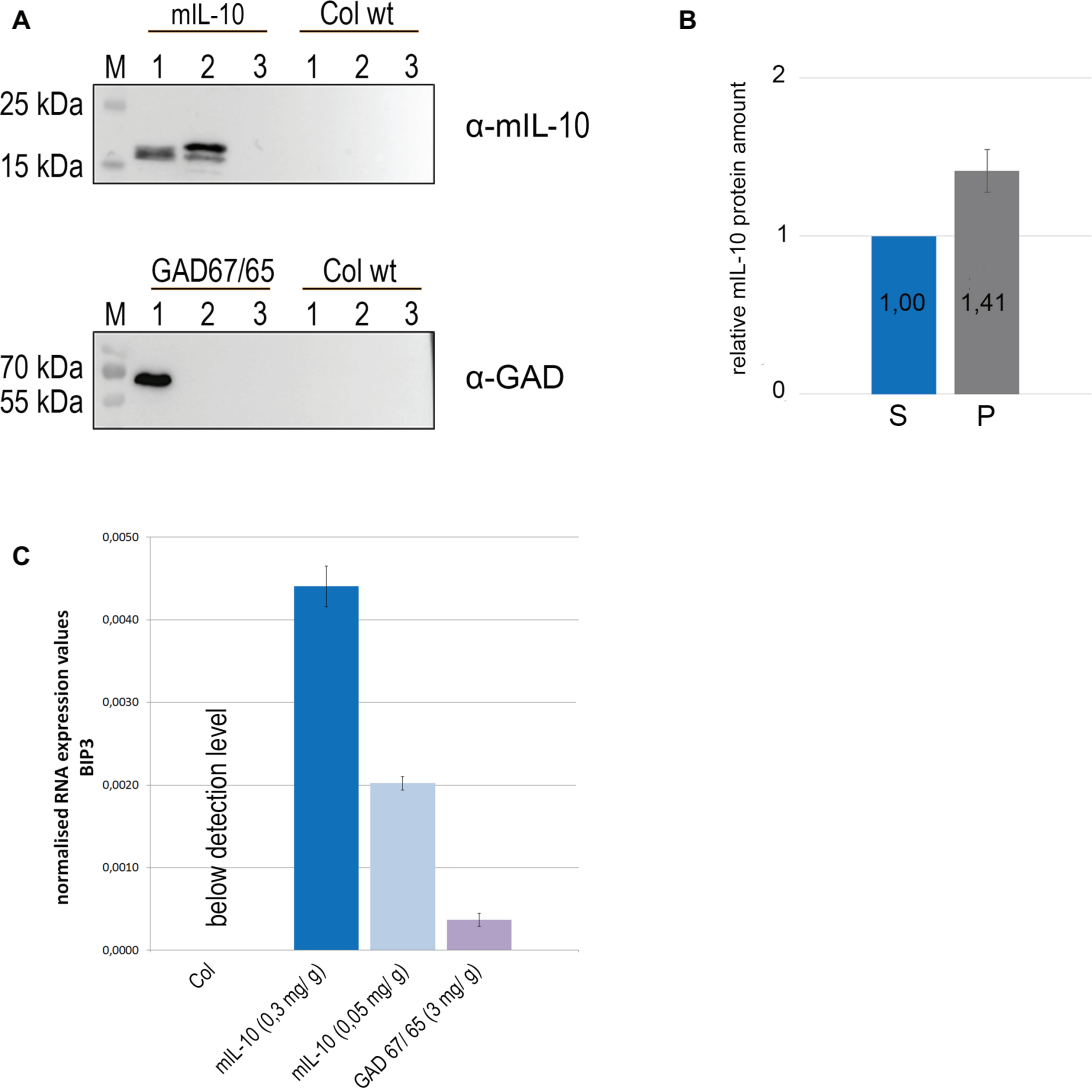
A large fraction of recombinant mIL-10 is insoluble under physiological conditions and its expression induces ER stress. **(A)** Sequential extraction of mIL-10, GAD67/65, and wild-type (Col wt) seeds followed by immunoblot analysis. Murine IL-10, GAD67/65, and wild-type (Col wt) seed extracts with saline buffer (lane 1), extraction of the pellet under reducing and denaturing conditions after three washes (lane 2), third saline wash before the re-extraction (lane 3). **(B)** Relative quantification of mIL-10 amounts in the soluble fraction (S) and in the pellet (P) shown in **(A)**. Quantification is based on five replicates (independent extractions of homozygous seed samples). Error bar depicts ± standard deviation of the mean. **(C)** Quantitative RT-PCR analysis of *BiP3* was performed using mature seeds of wild-type (Col) and transgenic lines expressing mIL-10 (at 0.3 or 0.05 mg/g, respectively) or GAD67/65. Transcript levels of *BiP3* were normalized to *LSM4* as internal control gene. Expression values are provided as E-ct *BIP3* / E-ct *LSM4*. Error bars depict ± standard deviation of the mean based on three replicates (measurement of three cDNAs derived from separate homozygous seed samples for each line). Plants compared in this experiment were grown at the same time under the same experimental conditions.

The multiple subcellular localization of mIL-10 might reflect protein fractions differing in solubility. Seeds were therefore extracted in saline buffer containing non-ionic detergent (0.1% Tween20), and the pellet was then re-extracted with the same buffer supplemented with 8 M urea and 5% 2-mercaptoethanol as denaturing and reducing agents. GAD67/65, which accumulates exclusively within PSVs at a level of 3 mg/g and does not induce ER-derived structures (Morandini et al., 2011), was analyzed under the same conditions as a traffic-competent control. A portion of the mIL-10 was recovered in the saline buffer and was detected by immunoblot at a molecular mass of approximately 19 kD, but more than half of the total amount remained insoluble and could only be recovered by re-extracting the pellet under denaturing and reducing conditions (Figures 2A,B). By contrast, GAD67/65 was completely extracted with the saline buffer, and no insoluble fraction was present in the pellet (Figure 2A). To determine whether the formation of disulfide bonds was responsible for the accumulation of insoluble mIL-10, the differential extraction was repeated only under denaturing conditions without reducing agent, followed by the extraction with an added reducing agent (Supplementary Figure S2). A major fraction of the protein was extracted only under reducing conditions, indicating that disulfide bonds might play a relevant role in the formation of the insoluble mIL-10 aggregates.

To determine whether the presence of insoluble protein aggregates of mIL-10 in the endomembrane system induces the unfolded protein response (UPR) we investigated the expression levels of the ER stress-responsive gene *BIP3* (Noh et al., 2003). In the UPR, genes encoding ER-resident molecular chaperones such as BiP are co-operatively induced to cope with misfolded proteins in the ER, and among three *BiP* genes in *Arabidopsis*, *BiP3* is most strongly regulated by bZIP60, a main transcription factor in UPR signaling (Iwata et al., 2008). We compared *BiP3* expression in seeds of two lines expressing mIL-10, in a wild-type control and in seeds producing GAD67/65 (Figure 2C). BiP3 was clearly induced in both transgenic lines expressing mIL-10 compared to wild-type. The line expressing GAD67/65 also showed an increase in *BIP3* transcripts, despite the normal structure of the ER and the absence of ER-derived protein aggregates, suggesting that the UPR induction is a general consequence of the expression of exogenous secretory proteins, but in mIL-10 expressing mature seeds the induction of BiP3 was five and 12 times higher, respectively (Figure 2C). Since even the highest levels of mIL-10 accumulation are markedly lower than those of GAD67/65, the data indicate a particularly high intrinsic propensity of miL-10 to induce ER-stress.

### Structures Containing mIL-10 Change the Morphology of the Endoplasmic Reticulum

**Figure 3:**
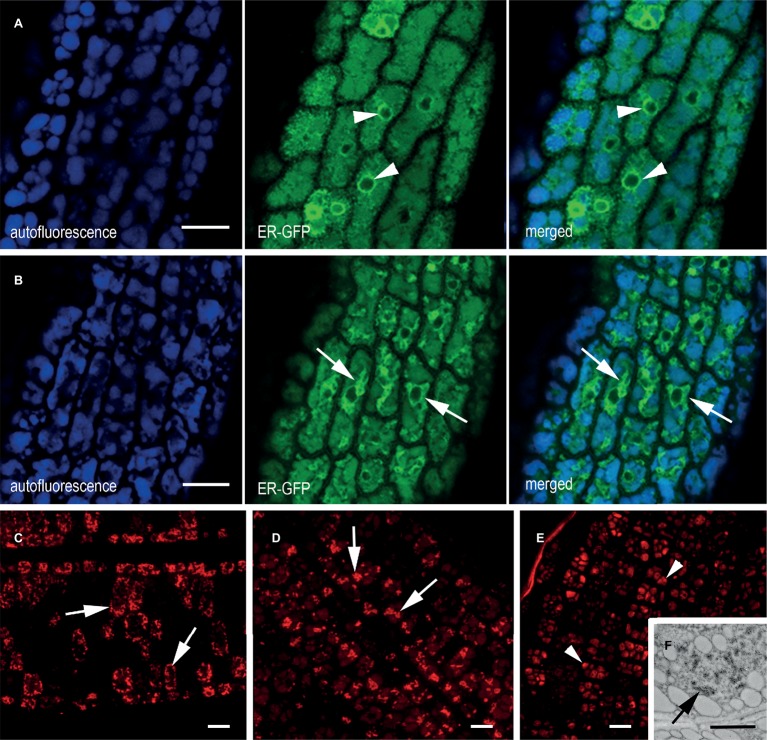
ER morphology in transgenic cotyledons of mature seeds. **(A,B)** *In vivo* confocal microscopy of cotyledon cells from **(A)** plants expressing ER-GFP, showing a clear ER network comprising the nuclear envelope (green channel, arrowhead) and **(B)** an F2 generation ER-GFP × mIL-10 cross showing an altered ER network (green channel, arrows). The blue channel reveals the autofluorescence of PSVs. **(C–E)** Immuno-localization of mIL-10 in fixed transgenic cotyledons showing clusters (arrows) in the lines with high **(C)** and moderate **(D)** production of mIL-10. The low expressing line shows labeling in the PSVs (**E**, arrowheads) but also in ER-derived structures (**F**, arrows). Bars = 10 μm **(A–E)**, 0.25 μm **(F)**.

The ER origin of the mIL-10 bodies was confirmed by crossing the best performing line expressing mIL-10 with an *A. thaliana* marker line expressing a green fluorescent protein fusion comprising a signal peptide for translocation into the ER, the GFP sequence, and the transmembrane and cytosolic domains of the type I integral membrane protein Cf-9 (ER-GFP). This chimeric trans-membrane protein is localized in the ER membrane *via* its cytosolic, C-terminal ER-localization motif KKXX and provides a tool to study ER morphology (Benghezal et al., 2000). Analysis of the ER-GFP marker line seeds confirmed the fine reticular pattern of the ER interspersed among the PSVs as well as the expected intense labeling of the nuclear envelope (Figure 3A, arrowheads). By contrast, the ER morphology in the mIL-10/GFP hybrid line formed patches and clusters rather than a network, in agreement with electron microscopy data, confirming that the recombinant mIL-10 caused the induction of ER-derived compartments throughout the tissue (Figure 3B, arrows). The intense signal of these novel structures indicates that they represent a large proportion of total ER.

The accumulation of mIL-10 in ER-derived structures was observed in all three lines we analyzed (Figures 3C–E; Supplementary Figure S3). In the best-performing line (0.3 mg/g mIL-10), we observed strong labeling of the clustered structures (Figure 3C), in agreement with electron and confocal microscopy data (Figures 1A–C, 3B). The strong fluorescence of these clusters did not allow to detect the probably much weaker signal from the low proportion of mIL-10 present in PSVs or the apoplast (Figure 3C). In the moderate-performance line, we also observed abundant ER-derived clusters within the cells (Figure 3D), but in the line with weak mIL-10 expression, the signal was mainly detected in the PSVs (Figure 3E). Nevertheless, immuno-electron microscopy of this line allowed to detect some clustered structures within the cytoplasm, indicating that even small amounts of recombinant mIL-10 can form aggregates within the ER (Figure 3F).

### The Structures Containing mIL-10 Are Spheroidal

**Figure 4:**
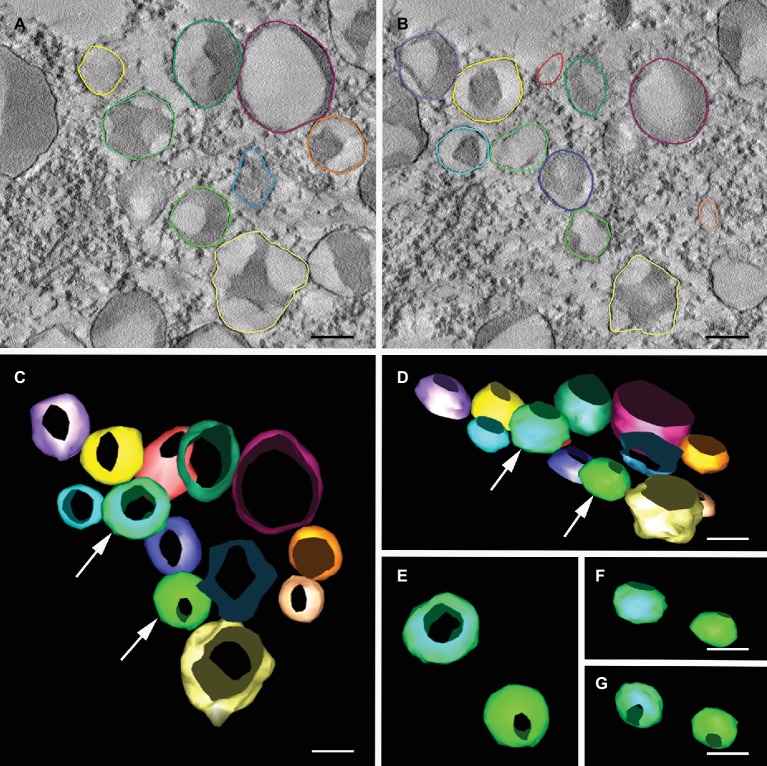
Electron tomography of Russell-like bodies containing mIL-10. Semi-thick sections (300 nm) from freeze-substituted, embedded transgenic seed embryos were imaged at 200 kV. **(A,B)** Cotyledon cell serial sections of transgenic *A. thaliana* seeds expressing mIL-10, showing the vesicles containing mIL-10 used for 3D modeling. **(C,G)** 3D model of the mIL-10-containing vesicles with the vesicles shown from different angles. Vesicles marked with an arrow **(C,D)** are shown from alternative angles **(E–G)** revealing their spheroidal shape and lack of connections. Bars = 0.25 μm.

The organization of the novel ER-derived bodies and their relationship with the rest of the ER was investigated by fixing transgenic *A. thaliana* cotyledons under low-temperature conditions for 3D imaging by electron tomography (Figures 4A,B). A different color was assigned to each of the bodies to improve the clarity of the tomographic model (Figures 4C–G). When observed from different angles, the spheroidal shape of the ER-derived bodies was clear, thus confirming that mIL-10 does not accumulate in dilated cisternae, saccules, or any kind of tubular or cylindrical structures (Figures 4E–G). Our observations therefore confirm that ER-derived structures containing mIL-10 are spheroidal Russell-like bodies.

### The mIL-10 Within Russell-Like Bodies Is Degraded More Slowly During Germination

**Figure 5:**
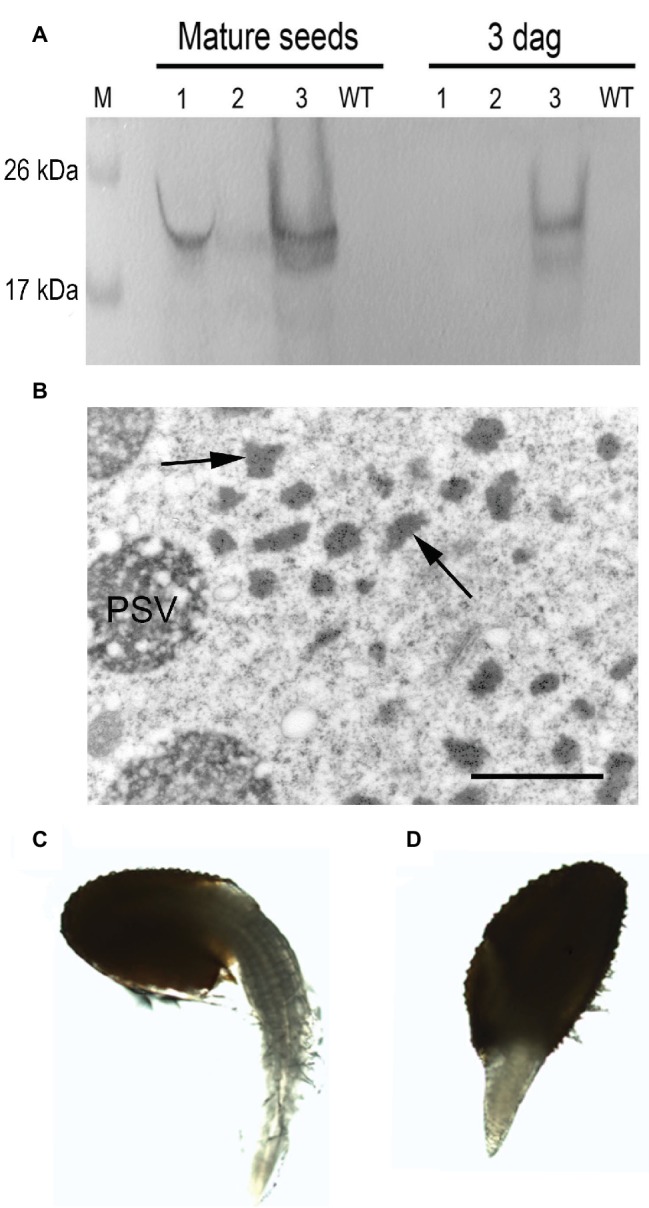
Analysis of transgenic mIL-10 seeds during germination. **(A)** Immunoblot analysis of mIL-10 seed extracts from mature dry seeds and from seeds 3 days after start of germination (3 DAG) Saline seed extract (lane 1), third pellet wash (lane 2), re-extraction of the pellet under reducing and denaturing conditions (lane 3). **(B)** Localization of mIL-10 in seeds at 3 DAG by immunoelectron microscopy. The ER-derived bodies containing mIL-10 remain unaltered (arrows), while the electrolucent areas within PSVs indicate that degradation of protein accumulated in this compartment has started. Bar = 1 μm. **(C)** Representative wild-type seed (Col), 3 DAG. **(D)** Representative transgenic seed (mIL-10), 3 DAG.

To explore the fate of the recombinant mIL-10 during germination, we extracted the soluble and the insoluble fractions from mature seeds before germination and at 3 days after germination (DAG). The mIL-10 soluble fraction was almost completely degraded at 3 DAG, whereas a large proportion of the insoluble fraction remained intact (Figure 5A). Many Russell-like bodies containing mIL-10 could still be observed by microscopy at the same time point (Figure 5B), whereas only a weak immunolocalization signal was detected in the remaining protein content of the PSVs (Figure 5B). This is consistent with the fact that upon germination, *Arabidopsis* endogenous storage proteins are degraded by newly synthesized proteases sorted to PSVs and indicates that the insoluble fraction accumulated in ER-derived structures does not readily enter in contact with proteases able to degrade it. Despite the distortion of the ER in cotyledon cells expressing mIL-10, the seeds are viable and germinated normally with only a slight delay compared to wild-type seeds (Figures 5C,D).

### The mIL-10 Is Sequestered Within Ectopic Prolamin Bodies

**Figure 6:**
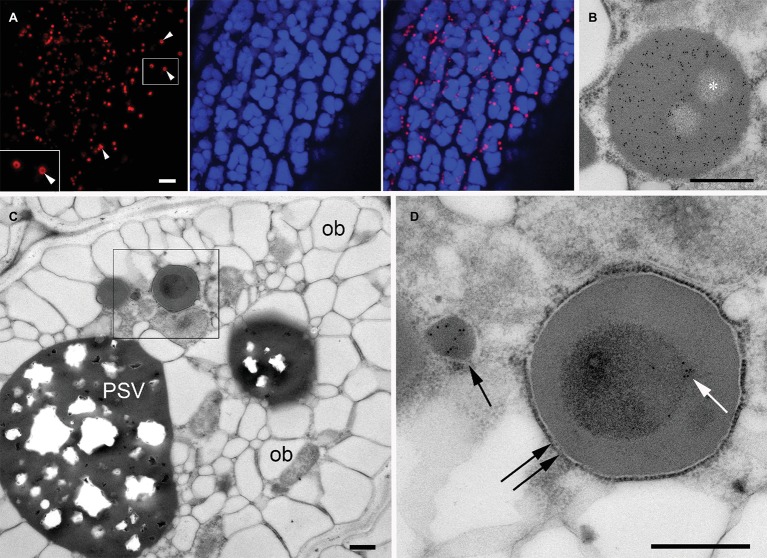
Ectopic DsRed-prolamin bodies in *A. thaliana* seed cotyledon cells. **(A,B)** DsRed bodies in the line expressing DsRed-zein alone. **(C,D)** DsRed bodies in the DsRed-zein × mIL-10 hybrid. **(A)** Confocal microscopy of *A. thaliana* cotyledons producing DsRed-zein bodies. Red channel: DsRed fluorescence. See the dark areas within some protein bodies (arrowhead). Blue channel: PSV autofluorescence. Right picture = overlay. **(B)** Electron microscopy of *A. thaliana* cotyledons producing DsRed-zein bodies: Localization of DsRed, see the abundant gold probes within the protein body and also the non-labeled areas (*). **(C,D)** Localization of mIL-10 in cotyledons of DsRed-zein × mIL-10 hybrid seeds. **(C)** Cotyledon cell overview, oil bodies (ob), protein storage vacuole (PSV). **(D)** Enlargement of the area outlined in **(C)**. See one labeled mIL-10 body (arrow) and the gold particles decorating the electron-dense material within the DsRed body (white arrow) delimited by a ribosome-studded membrane (double arrow). Bars = 5 μm **(A)**, 0.5 μm **(B–D)**.

PBs containing large insoluble prolamin polymers are typically found in seeds of Poaceae, including cereals (Khoo and Wolf, 1970; Larkins and Hurkman, 1978), whereas *A. thaliana* seeds mainly produce PSV-located globulins (cruciferin) and albumins but no prolamins (Hinz et al., 2007) and therefore do not generate such ER-derived protein bodies. However, it is possible to induce the formation of ectopic ER-derived protein bodies by expressing heterologous prolamin sequences (Bagga et al., 1995; Coleman et al., 1996; Mainieri et al., 2004; Llop-Tous et al., 2010). The N-terminal domain of the 27 kDa γ-zein (a major storage prolamin in maize endosperm) is sufficient to confer the ability to form PBs when fused to other proteins (Mainieri et al., 2004; Torrent et al., 2009). Therefore, we fused this N-terminal domain to the fluorescent protein DsRed and generated transgenic *A. thaliana* plants expressing this construct. As expected, transgenic seeds expressing the chimeric protein formed abundant red fluorescent protein bodies distinct from PSVs in the cytoplasm of the cotyledonary cells (Figures 6A,B). The red fluorescent PBs were spherical, with a diameter of 1–1.5 μm. In some of the DsRed bodies, a dark central area was observed by confocal microscopy (Figure 6A, arrowheads). Consistent with the fluorescence images, in electron microscopy DsRed bodies have a uniform electron density and homogeneous labeling with anti-DsRed rabbit serum, except for well-defined areas that are more electrolucent and devoid of gold particles (Figure 6B, asterisk). These areas indicate that that the DsRed-bodies often resemble spherical shells with unknown content in their cavities.

To investigate the behavior of mIL-10 in the presence of prolamin-induced, ER-derived PBs, we crossed the DsRed-zein body line and the best-performing line producing mIL-10. Russell-like bodies containing mIL-10 were less abundant in the hybrid seeds than in lines solely expressing mIL-10, and the cotyledon cells were similar in structure to wild-type cells except for the presence of the DsRed bodies (Figure 6C, compared to Figure 1C). However, the appearance of the DsRed bodies was different in the hybrid line and the lines expressing DsRed-zein alone. The bodies in the hybrid line were often filled with an inner core of more electron-dense material, which was labeled by the anti-mIL-10 serum. These experiments indicated that mIL-10 was located within the DsRed bodies and partially filled the central areas of the prolamin-induced DsRed PBs (Figure 6D, and compared with Figure 6B).

### Russell-Like Bodies Are Also Induced by Other Recombinant Proteins

**Figure 7:**
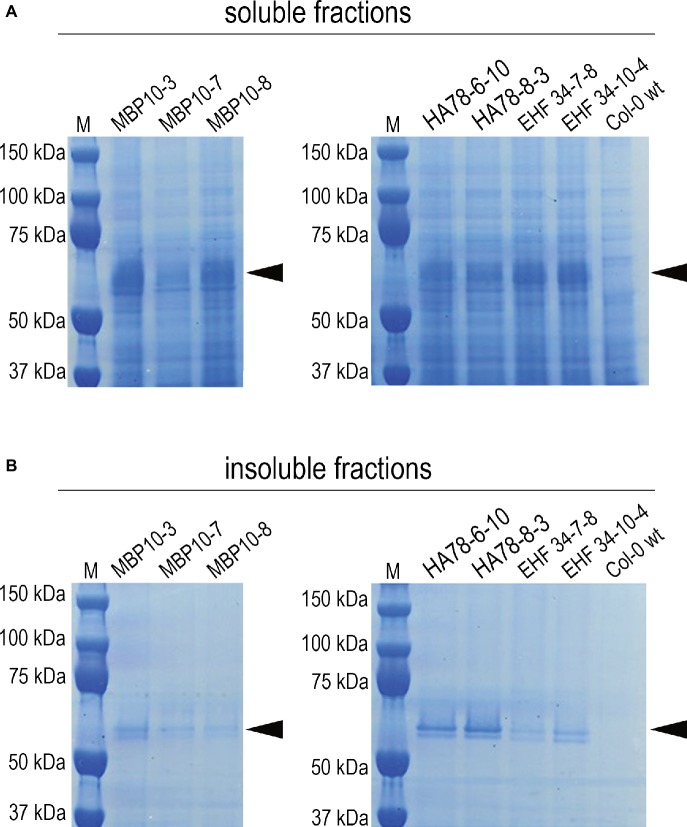
Soluble and insoluble antibody fractions from mature seeds of different *A. thaliana* lines expressing scFv-Fc antibodies MBP-10, HA78, or EHF34, respectively (Van Droogenbroeck et al., 2007). Bands corresponding to the scFv-Fc antibodies with a molecular mass around 55 kD are indicated by an arrow. After extracting *A. thaliana* seeds in saline buffer containing non-ionic detergent **(A)**, the pellets were re-extracted with the same buffer supplemented with 8 M urea and 5% 2-mercaptoethanol as denaturing and reducing agents **(B)**. About 30 μg of total soluble protein was loaded on SDS-PAGE (reducing conditions, 4–12% Bis-Tris polyacrylamide gel), followed by Coomassie staining.

It has been previously reported that some single chain Fv-Fc antibodies produced in *A. thaliana* seeds were partially localized in ER-derived vesicles (Van Droogenbroeck et al., 2007; Loos et al., 2011). Even though the shape of these ER-derived structures was not investigated in detail, their morphology suggests they are also Russell-like bodies. However, protein solubility was not investigated in these studies. We therefore re-examined seeds expressing scFv-Fc antibodies MBP-10, HA78, and EHF34 at levels between 7 and 13% TSP (Van Droogenbroeck et al., 2007). Following saline extraction (Figure 7A), we re-extracted the pellet under denaturing and reducing conditions and the presence of a significant antibody fraction obtained from the pellet was confirmed by SDS/PAGE (Figure 7B) and by immunoblot (Supplementary Figure S4).

## Discussion

The aim of this study was to characterize the endomembrane compartments formed in response to the accumulation of recombinant mIL-10 in *A. thaliana* cotyledons. Immunofluorescence microscopy and electron tomography showed that the ER-derived bodies containing insoluble mIL-10 were spheroidal membrane-bound structures. This is strongly reminiscent of the Russell bodies found in mammalian cells, containing condensed proteins that can only be solubilized with organic solvents, reducing agents or other denaturing conditions (Kopito and Ron, 2000; Kopito and Sitia, 2000). Consistent with our data, it has been shown that RBs are not simply expanded ER membranes, but distinct membrane-surrounded entities with limited connection to the rest of the ER network, possibly *via* narrow tubules (Kimura et al., 2015). It is not fully understood that proteins trigger the formation of RBs in mammalian cells, and under which conditions. For example, RBs can be induced in lymphoid and non-lymphoid cells by the expression of mutant IgM or other immunoglobulin isotypes, and in this process, the formation of the RBs can be influenced by manipulating the rate of heavy chain synthesis and degradation (Valetti et al., 1991; Kaloff and Haas, 1995). Similarly, Russell-like bodies formed in the transgenic *A. thaliana* lines containing low, moderate, and high levels of mIL-10. However, the structures were less abundant in the line with the weakest mIL-10 expression, where part of the cells lacked the ER-derived bodies altogether.

GAD67/65 and mIL-10 were expressed using the same regulatory sequences, but GAD67/65 did not form condensed structures within the ER despite being present at more than 10-fold higher levels than mIL-10. This indicates that the induction of ER-derived bodies is not due to a general saturation of the protein export capacity of the ER. The biogenesis of these ER-derived structures instead appears to depend on protein-specific properties, such as the intrinsic propensity for condensation or polymerization, or the frequency of misfolding, as also indicated by our observation that in mature seeds expression of the specific ER stress marker BiP3 is induced to much higher levels by mIL-10 than by GAD67/65. The relative rates of protein folding, misfolding, and exit from the ER are indeed protein specific and determine the destiny of a given protein along the secretory pathway (Wiseman et al., 2007). In the chimeric protein GAD67/65, the N-terminal domain of GAD67 leads to increased stability of the protein when targeted to the ER, and it has been speculated that this may be due to a positive influence on protein folding (Avesani et al., 2010). It is also reasonable to hypothesize that the propensity for polymerization/aggregation of a given protein plays a role in avoiding both exit from the ER and degradation by quality control (Vitale and Ceriotti, 2004; Mainieri et al., 2014). In addition, cell specific factors could also influence the intrinsic propensity for condensation, as determined in mammalian cells (Mossuto et al., 2015) and indicated by the observation that when mIL-10 was transiently expressed in agroinfiltrated *N. benthamiana* leaves, it did not form accretion bodies (Westerhof et al., 2012). In line with this, the RB phenotype in mammalian cells is considered to originate from an interplay between the intrinsic condensation propensity of a recombinant protein and various extrinsic parameters of the cellular environment (Hasegawa et al., 2014). This is underlined by the observations that absolute insolubility is not a strict requirement for any protein that is naturally part of protein bodies (Mainieri et al., 2018) or is able to form Russell bodies (Hasegawa et al., 2014).

Interleukin-10 is a homodimer. Two conserved intrachain disulfide bonds play an important role in folding of the monomer. The murine IL-10 sequence contains a fifth, unpaired Cys residue at position −11 from the C-terminus, which is absent from the highly similar human IL-10 and is therefore not involved in assembly or biological activity (Windsor et al., 1993). This unpaired residue negatively affects correct disulfide bond formation during *in vitro* refolding of mIL-10 produced in *Escherichia coli* (Kunze et al., 2014) and therefore could lead to the formation of incorrect disulfide bonds and perhaps aggregation in conditions where correct folding is inefficient due to high protein synthesis. Previously, it has been shown that human IL-10 produced in rice seeds was detergent-insoluble and localized in ER-derived prolamin-containing bodies (Fujiwara et al., 2010; Yang et al., 2012) suggesting that a portion of the recombinant protein interacted with rice prolamins through intermolecular disulfide bonds (Yang et al., 2012). However, the Russell-like bodies containing mIL-10 in *A. thaliana* formed in the absence of prolamins and endogenous prolamin bodies, indicating an intrinsic ability of mIL-10 to condense and induce ER-derived structures *de novo*.

Single chain Fv-Fc antibodies produced in *A. thaliana* seeds also partially localized in ER-derived structures (Van Droogenbroeck et al., 2007; Loos et al., 2011), and for each of these scFv-Fc antibodies, we identified a significant portion that was insoluble in non-ionic detergent. Remarkably, the scFv-Fc molecules consisted of the variable antibody regions fused to a human IgG1 Fc domain including the hinge, CH2 and CH3 domains, but lacking the CH1 domain. This is reminiscent of a routine procedure that is commonly used to induce RBs in mammalian cells and involves the expression of mutant immunoglobulin isotypes lacking the CH1 domain that binds to BiP during normal antibody assembly (Munro and Pelham, 1986; Mattioli et al., 2006). Heavy chains lacking this domain apparently have a tendency to form insoluble aggregates in the ER, probably because they bind less BiP and do not benefit sufficiently from its chaperone activity (Mattioli et al., 2006). It is important to note, however, that antibody clones that were found to induce RBs in mammalian cells were by no means defective in terms of their folding stability, antigen binding, and *in vitro* biologic activity (Hasegawa et al., 2017). Functionality has also been demonstrated for the soluble fractions of the seed-produced recombinant proteins investigated in the present study (Van Droogenbroeck et al., 2007; Morandini et al., 2011).

Although it is not fully understood that protein structural features trigger the formation of RBs in mammalian cells, and under which conditions, the deposition of insoluble proteins in these specialized compartments seems to alleviate the negative effects of such proteins on the endomembrane system (Valetti et al., 1991; Granell et al., 2008). The protective effects of Russell bodies have been reported in neurons and myeloma cells (Granell et al., 2008; Ito et al., 2012), which remain viable and undergo mitosis despite the presence of massive protein aggregates (Weiss et al., 1984; Alanen et al., 1985; Valetti et al., 1991). Similarly, we found that the *A. thaliana* cotyledon cells remained viable despite the numerous protein accretions, and seeds containing Russell-like bodies germinated normally with only a short delay compared to wild-type seeds.

Detergent-insoluble storage proteins (prolamins) in the Poaceae also take advantage of controlled insolubilization/polymerization to escape degradation in the ER and they accumulate in protein bodies that share some features with Russell bodies (Vitale and Ceriotti, 2004). Most prolamins form large PBs because of hydrophobic interactions and/or inter-chain disulfide bonds (Pedrazzini et al., 2016), and this mechanism is particularly well studied in maize, where the ability of the 27 kDa γ-zein to induce the formation of PBs has been mainly attributed to its N-terminal half, including seven Cys residues and an amphipathic proline-rich sequence stretch (Geli et al., 1994; Pompa and Vitale, 2006; Llop-Tous et al., 2010). We fused this N-terminal region of 27 kDa γ-zein to DsRed so that ectopic ER-derived fluorescent protein bodies were induced in *A. thaliana* seeds. We crossed a transgenic line expressing this fusion protein to another transgenic line expressing mIL-10, to verify the behavior of mIL-10 in the presence of prolamin-induced protein bodies. The hybrid transgenic line predominantly contained one population of protein bodies in which mIL-10 inclusions were embedded within the prolamin-containing matrix. These data indicate that both types of accreting proteins were together selected out of secretory traffic by the formation of separate, membrane-bound compartments, which likely prevent them from negatively interfering with the folding and transport of soluble proteins. The ordered distribution of different proteins in the same ER-derived body resembles the structure of maize PBs: α-zeins occupy most of the core and are surrounded by a thin layer constituted mainly of γ-zeins and the less abundant β-zein, which remain in contact with the luminal face of the ER membrane (Lending and Larkins, 1989; Kogan et al., 2004). The mIL-10 aggregates thus appeared to be able to replicate the natural behavior of α-zeins in the structure of a maize PB. This indicates that the γ-zeins, which are primarily responsible for the correct organization of mature maize PBs (Wu and Messing, 2010), generate a central volume that can accommodate other protein assemblies such as α-zeins or mIL-10. It has been reported that soluble proteins in the secretory pathway can also be passively sequestered into PBs induced by fusion tags based on γ-zein (Joseph et al., 2012), elastin-like polypeptide (ELP), or hydrophobin (HFBI; Saberianfar et al., 2015). Interestingly, however, zein-induced PBs did not co-localize with either ELP- or HFBI-induced protein bodies (Saberianfar et al., 2016), indicating that certain molecular interactions may be necessary for the formation of multi-component PBs and that not all accretions formed within the ER have similar physical interactions with each other.

In conclusion, we have provided evidence that Russell-like bodies occur in plant seeds in a similar manner as in mammalian and yeast production hosts when confronted with large quantities of recombinant protein. It remains to be investigated in detail if seeds are more prone to the formation of Russell-like bodies than other plant organs. The appearance of visually identifiable protein bodies upon reporter gene expression in tobacco leaves has been reported, but protein solubility was not investigated in these studies (Gutierrez et al., 2013; Saberianfar et al., 2015). It was further suggested that the formation of the observed protein accretions is a concentration-dependent mechanism occurring at recombinant protein levels above 0.2% of total soluble protein, regardless of the presence of a fusion tag such as HFBI or ELP (Saberianfar et al., 2015). This is in agreement with our data, which, however, suggest that the threshold level for Russell-like body formation differs between recombinant proteins. Considering that the threshold level in mammalian systems also depends on host-specific factors (Mattioli et al., 2006), this may offer an opportunity for host plant engineering to optimize the yield of functional, soluble protein.

## Data Availability

All datasets generated for this study are included in the manuscript and/or the Supplementary Files.

## Author Contributions

ES, EA, and AV contributed conception and design of the study and wrote sections of the manuscript. EA and DP designed and carried out electron microscopy and tomography experiments. VI and EA designed and performed confocal analyses. JH designed and analyzed the ER stress markers. LA, LB, MP, TR, and FM generated and characterized the *Arabidopsis* lines. AD and TM contributed and analyzed antibody producing plants. All authors contributed to manuscript revision, read, and approved the submitted version.

### Conflict of Interest Statement

The authors declare that the research was conducted in the absence of any commercial or financial relationships that could be construed as a potential conflict of interest.
