# Supplementary material for: Russell-Like Bodies in Plant Seeds Share Common Features With Prolamin Bodies and Occur Upon Recombinant Protein Production
Source: Front Plant Sci. 2019 Jun 26;10:777. doi: 10.3389/fpls.2019.00777 (PMC6611407; doi:10.3389/fpls.2019.00777)
Supplement: Supplementary file 1 [file Data_Sheet_1.pdf]

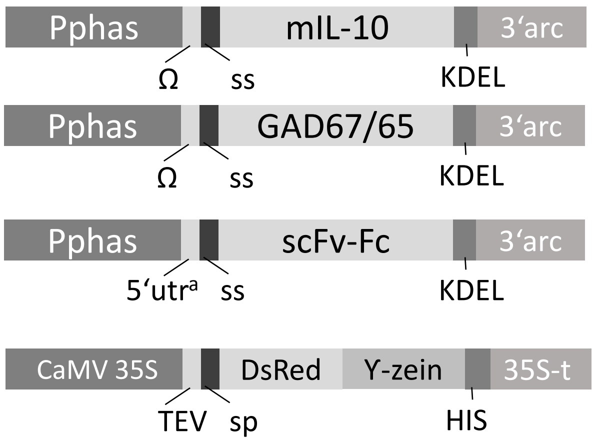

Figure S1. Schematic overview of the transgene cassettes used in the study. Pphas, β-phaseolin gene promoter (−1 to −1,470; GenBank accession no. J01263); 5′ utr[a], 5′ UTR of *arc5-I* gene (13 bp; part of GenBank accession no. Z5020); 3′ arc, 3′-flanking regulatory sequences of the *arc5-I* genomic clone (3,900 bp; part of GenBank accession no. Z50202); ss, signal peptide of the *Arabidopsis* 2S2 seed storage protein gene; scFv-Fc, scFv-Fc coding sequence; Ω leader, 5′ untranslated region (UTR) of tobacco mosaic virus; KDEL, ER retention signal; TEV, *Tobacco etch virus* 5′-untranslated region; sp, plant codon-optimized signal peptide sequence derived from a murine antibody; DsRed, tetrameric DsRed; HIS, polyhistidine tag; CaMV35S and 35S-t, cauliflower mosaic virus 35s enhanced promoter and terminator; ϒ-zein, residues 4–93 of the mature 27 kDa γ-zein protein. Not drawn to scale. (Van Droogenbroeck et al., 2007; Morandini et al., 2011; Hofbauer et al., 2014)

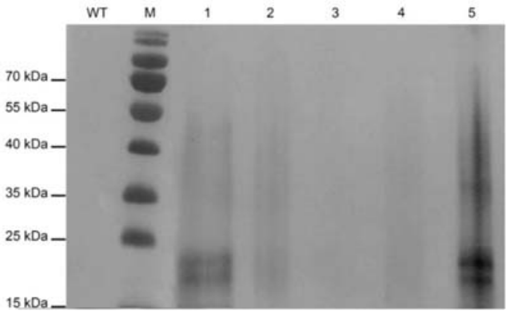

Figure S2. Sequential extraction of mIL-10. Salt extract (lane 1), third wash (lane 2), re-extraction of the pellet under denaturing conditions (25 mM Tris-HCl pH 7.8, 1.6% SDS; lanes 3 and 4), re-extraction of the pellet under reducing and denaturing conditions (25 mM Tris-HCl pH 7.8, 1.6% SDS, 100 mM DTT; lane 5), wild type (WT).

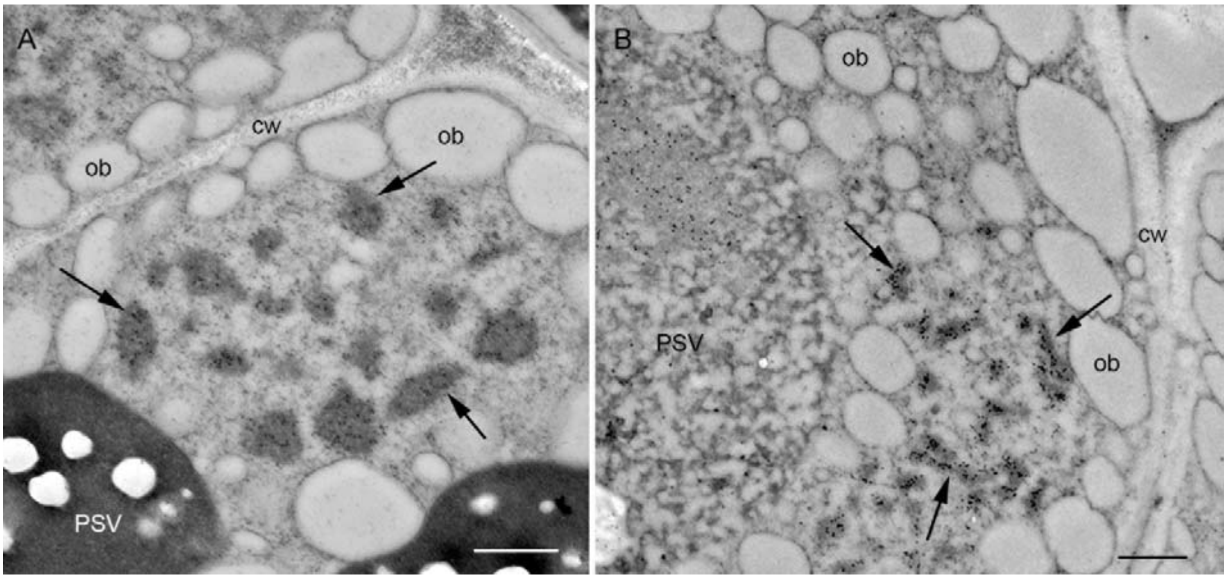

Supplemental Figure S3. **Localization of mIL-10 in A. thaliana cotyledon cells in lower expressing lines**. Murine IL-10 accumulates in Russell-like bodies in lines expressing 0.14 mg/g (A, arrows). Even in low expressing lines (0.05 mg/g), mIL-10 can be found in ER-derived structures (B, arrows), as well as in PSVs. Cell wall (cw), oil bodies (ob). Bars 0,5 µm

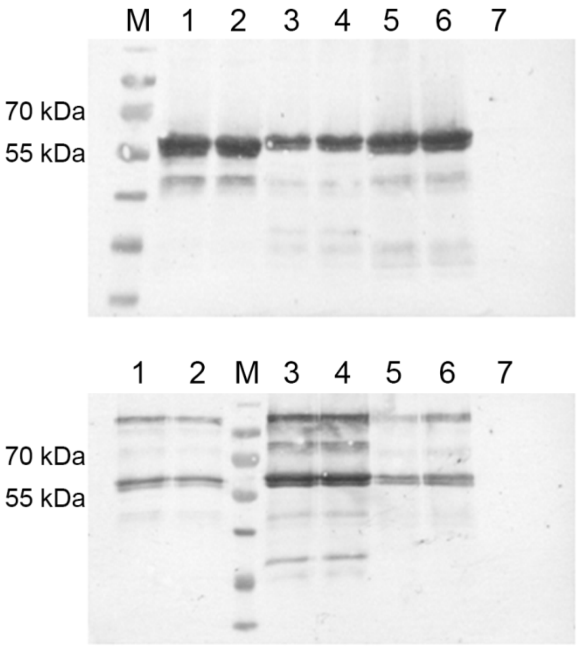

Supplemental Figure S4. Immunoblot of soluble and insoluble antibody fractions from seeds of *A. thaliana* lines expressing scFv-Fc antibodies MBP-10, HA78 or EHF34, Upper panel: soluble fraction using saline buffer. Lower panel: pellet re-extracted under reducing and denaturing conditons. Anti-Human IgG serum was used for detection. M- Marker, 1-MBP10-31-7, 2- MBP10-39-7-1, 3- HA78-6-8, 4-HA78-8-3, 5-EHF-34-10-4, 6-EHF-34-10-4, 7- WT
